# DNA methylation patterns and gene expression associated with litter size in Berkshire pig placenta

**DOI:** 10.1371/journal.pone.0184539

**Published:** 2017-09-07

**Authors:** Jung Hye Hwang, Sang Mi An, Seulgi Kwon, Da Hye Park, Tae Wan Kim, Deok Gyeong Kang, Go Eun Yu, Il-Suk Kim, Hwa Chun Park, Jeongim Ha, Chul Wook Kim

**Affiliations:** 1 Swine Science and Technology Center, Gyeongnam National University of Science & Technology, Jinju, South Korea; 2 Department of Animal Resource Technology, Gyeongnam National University of Science & Technology, Jinju, South Korea; 3 Dasan Pig Breeding Co., Namwon, South Korea; Universitat de Lleida, SPAIN

## Abstract

Increasing litter size is of great interest to the pig industry. DNA methylation is an important epigenetic modification that regulates gene expression, resulting in livestock phenotypes such as disease resistance, milk production, and reproduction. We classified Berkshire pigs into two groups according to litter size and estimated breeding value: smaller (SLG) and larger (LLG) litter size groups. Genome-wide DNA methylation and gene expression were analyzed using placenta genomic DNA and RNA to identify differentially methylated regions (DMRs) and differentially expressed genes (DEGs) associated with litter size. The methylation levels of CpG dinucleotides in different genomic regions were noticeably different between the groups, while global methylation pattern was similar, and excluding intergenic regions they were found the most frequently in gene body regions. Next, we analyzed RNA-Seq data to identify DEGs between the SLG and LLG groups. A total of 1591 DEGs were identified: 567 were downregulated and 1024 were upregulated in LLG compared to SLG. To identify genes that simultaneously exhibited changes in DNA methylation and mRNA expression, we integrated and analyzed the data from bisulfite-Seq and RNA-Seq. Nine DEGs positioned in DMRs were found. The expression of only three of these genes (*PRKG2*, *CLCA4*, and *PCK1*) was verified by RT-qPCR. Furthermore, we observed the same methylation patterns in blood samples as in the placental tissues by PCR-based methylation analysis. Together, these results provide useful data regarding potential epigenetic markers for selecting hyperprolific sows.

## Introduction

Litter size is an economically important trait in the pig industry [1] and a great deal of effort has been devoted to improving this trait. A number of factors, including physiological components as well as environment, diet, and strain, have been shown to affect litter size. Different selection strategies can be implemented to emphasize various physiological components that determine litter size, such as ovulation rate, embryo survival, fetal survival, and placental efficiency [2, 3]. The placenta and uterus may play central roles in prolificacy [4, 5]. Placental efficiency is a critical component of uterine capacity in pigs, and maximizing this factor can help increase litter size [6].

DNA methylation, an important epigenetic modification, involves the addition of a methyl group to the fifth carbon of cytosine (C) in CpG dinucleotides, forming 5-methylcytosine, and it is catalyzed by DNA methyltransferases [7]. Many recent studies have examined the genome-wide methylation profiles of livestock phenotypes that are associated with disease resistance, milk production, and reproduction [8–11]. DNA methylation affects the expression of many genes that are critical to reproduction traits [12–14]. Furthermore, Kwon *et al*. recently identified differentially expressed genes (DEGs) via transcriptomic analyses of litter size in pig placentas and suggested that the *IL-6* and *LIPG* genes play important roles in increasing litter size by increasing nutrition supply through the placenta [15]. However, do date no studies have investigated how the DNA methylation patterns in pig placental tissue are associated with litter size.

Therefore, we performed a genome-wide examination of the differences in DNA methylation and gene expression in the placental tissues of two groups of pigs with smaller (SLG) and larger (LLG) litter sizes. Our results provide useful information on the reproductive phenotype of individual pigs and could help make selection in the livestock industry more cost-effective.

## Materials and methods

### Ethics statement

The experimental protocols for this study were approved by the Gyeongnam National University of Science and Technology Institutional Animal Care and Use Committee (Permit Number: 2105–5).

### Animal and placenta collection

All of the Berkshire sows used in this study were reared under the same environmental conditions (Dasan Pig Breeding Co., Namwon, Korea). Animals were given the same commercial diet and water *ad libitum*. To classify the sows by litter size, they were divided into two groups with low and high estimated breeding value (EBV), and three sows per group were selected. SLG had EBV ≤ 0.75 and average litter size < 7, while LLG had EBV ≥ 0.75 and average litter size > 12. There were no significant differences in other characteristics such as age, body weight, and total parity, between the two groups (data not shown). The placentas were collected immediately after delivery from tree sows in each group, all in fourth parity. Samples were excised from the maternal side of the placenta, 2 cm from the site of umbilical cord insertion, and free of maternal decidua. The collected placental tissues were rapidly frozen in liquid nitrogen. Genomic DNA and mRNA was extracted, and pooled for sequencing analyses. Pooled sampling is cost-effective but can mask some variation, so further validation of candidate genes was performed on individual samples from three additional sows. The collection and storage of these additional samples were performed in the same way as described above, except for the pooling process.

### Genomic DNA isolation

Genomic DNA was separated from the recovered placentas of animals in each group using a Wizard Genomic DNA Purification Kit (Promega, Madison, WI). DNA purity was assessed using a NanoDrop^™^ Spectrophotometer (Thermo Fisher Scientific, Waltham, MA) and DNA concentration was measured using a Quant-iT^™^ dsDNA Broad Range Assay kit (Life Technologies, Rockville, MD).

### Genome-wide bisulfite sequencing

Genomic DNA (≥ 6 μg) was fragmented by sonication to approximately 100~300 bps, followed by end-repair. Fragmented DNA was bisulfite-converted using the EZ DNA Methylation-Gold^™^ Kit (Zymo Research, Orange, CA) according to the manufacturer’s instructions. Bisulfite treatment converted unmethylated cytosines to uracils while leaving methylated cytosines unchanged. Bisulfite-converted DNA was quantified using a Quant-iT^™^ dsDNA High Sensitivity Assay Kit (Life Technologies) on an Agilent 2100 Bioanalyzer (Agilent Technologies, Inc., Santa Clara, CA) and was used as a template for PCR amplification. After quantitative PCR (qPCR) amplification, the resulting libraries were subjected to paired-end sequencing with a 100-bp read length using the Illumina HiSeq 2500 platform (Illumina, San Diego, CA).

### DMRs analysis

The raw sequencing reads were cleaned by removing adaptor sequences, and reads in which the percentage of unknown bases was greater than 10% or low-quality reads were filtered out to keep only the high-quality reads. The clean reads were mapped to the pig reference genome (Sscrofa v10.2) using Bismark (version 0.9.0) with two allowed mismatches [16], and the reads that overlapped polymorphic sites were distinguished by comparison with the positions of all known single nucleotide polymorphisms. Methylated cytosines were extracted from aligned reads using the Bismark methylation extractor with the standard parameters. The methylation level of a C within an aligned read was determined by calculating the ratio of the number of reads that contained a methylated C at the location to the number of all reads that covered the location. Each sequence content of the methylated C was separately considered as CG, CHG, or CHH, where H is A, C, or T. For analysis of the average methylation levels in different genomic regions, including genes (promoter, 5'-untranslated region (UTR), 3'-UTR, coding sequences, and introns), CpG islands, and transposable elements, the average methylation levels for all regions were calculated and plotted using R (version 3.0.2) (S1 Fig). DMRs between the two groups were predicted using CpG_MP with the default parameters (length, CG content, and CpG ratio) [17], and their genomic distributions were investigated. Furthermore, we identified differentially methylated genes (DMGs) when a DMR and a specific gene function element (such as promoters) overlapped using the University of California Santa Cruz Genome Browser Database. For functional annotation of genes, Gene Ontology (GO) analysis was performed using DAVID Bioinformatics Resources v6.7 (http://david.abcc.ncifcrf.gov/).

### RNA isolation and sequencing

Total RNA was isolated from the pooled placentas of the three Berkshire pigs in each group using TRI-reagent (Molecular Research Center, Cincinnati, OH). The quality of total RNA was measured using an Agilent 2100 Bioanalyzer (Agilent Technologies, Santa Clara, CA). To identify DEGs, RNA sequencing (RNA-Seq) libraries were constructed using a TruSeq RNA Sample Prep Kit (Illumina) as described previously [15]. Expression levels were quantified using HTSeq-count (ver. 0.5.4p3) [18] and DEGs were determined using TCC [19] with the negative binomial statistical test available in two R packages iDEGES/edgeR and cutoff values of *p* < 0.05 and 1.5-fold change. Cutoff values were calculated as fragments per kilobase of exon per million fragments mapped (FPKM) and log2 (FPKM) values for genes in each group. Furthermore, DEGs related to fecundity were identified using GO annotation and KEGG pathway analysis.

### Analysis of DEGs positioned in DMRs between the two groups

To identify DEGs positioned in DMRs, we first selected the DMGs by a difference rate of methylation level > 30% between SLG and LLG. Subsequently, the DEGs from selected genes were detected using an RNA-Seq DEG analysis cut-off of *p* < 0.01 and *q* < 0.05.

### Real-time quantitative PCR (RT-qPCR) for DEG verification

RT-qPCR was performed to further verify expression of the selected DEGs. Total RNA of placental tissue was extracted with TRIzol reagent (Molecular Research Center) and then reverse transcribed using Superscript II (Life Technologies) with a reverse primer specific for each gene (Table 1). The amplification of cDNA was performed using 40 cycles at 94°C for 5 s and 60°C for 10 s, as described previously [15]. Melting curve analysis was performed at the end of PCR for 5 s from 60°C to 95°C to identify unique PCR products amplified during the reaction. Peptidylprolyl isomerase A (*PPIA*) was used as a reference gene [20] and the 2^−*ΔΔCT*^ method was used to analyze the changes in relative gene expression from the qPCR experiments. The significance of differences was analyzed using Student’s t test. These reactions were performed at least three times.

**Table 1:** Primer sequences used in RT-qPCR.

Gene symbol	Description	Accession No.	Primer sequences (5'→ 3')	Product size (bp)
*COL18A1*	Collagen, type XVIII, alpha 1	ENSSSCG0 0000030160	F: CTTCCTCGCCTGGTTCTTCT R: AGCCGGCCCAATGAA AC	196
*ABP1*	Amiloride binding protein 1	ENSSSCG0 0000016442	F: AATTTCTACGCGGGGCTCAA R: GGTCGACGCGGTAATTCAC	241
*SLC45A4*	Solute carrier family 45, member 4	ENSSSCG0 0000005930	F: CCTACAACTCGGGCGTGAA R: CTTGATGTCGTGGTACTGGC	281
*PRKG2*	Protein kinase, cGMP-dependent, type II	ENSSSCG0 0000009250	F: GGTTCCGTGAAACCCAAACA R: CACCACATCCTGAAGCTTGTT	249
*CLCA4*	Chloride channel, calcium activated, family member 4	ENSSSCG0 0000006932	F: TGCTATAGACCCTGCTGTGC R: TGCAACAATGATATCAGCGT	211
*PCK1*	Phosphoenolpyruvate carboxykinase 1	ENSSSCG0 0000007507	F: TCCGACCTTCCTTGACCATC R: GCCTCTTGATGACACCCTCT	283
*SLC6A19*	Solute carrier family 6 (neutral amino acid transporter), member 19	ENSSSCG0 0000017120	F: ATCCATCCGGCGCTGAAG R: TCGCGGTACCAGAAGTAGTC	218
*EPS8L3*	Epidermal growth factor receptor kinase substrate 8-like protein 3	ENSSSCG0 0000006820	F: TGGATCACAGGACCTCTACC R: ATGACGCCCTAGAACCCAAA	153
*F10*	Coagulation factor X	ENSSSCG0 0000009558	F: CAAGTCCTGCATCTCCACAG R: CACTATCCGGACCAGGCTG	226

### PCR-based methylation analysis

Blood samples were collected from the three sows used to obtain placental tissues in each group for RNA-Seq and genomic DNA was isolated using a Wizard Genomic DNA Purification Kit (Promega, Madison, WI) according to the manufacturer’s instructions. For cytosine methylation analysis, the DNA was digested to completion using a pair of methylation-sensitive isoschizomers, *Hpa*II and *Msp*I (NEB, Beverly, MA) and primers were designed flanking the *Hpa*II/*Msp*I sites. PCR was carried out as follows: 5 minutes at 94°C, followed by 35 or 40 cycles of 30 s at 94°C, 30 s at 60 C, and 30 s at 72°C, with a final elongation step of 7 minutes at 72°C.

## Results

### DNA methylation profile of placenta

To generate DNA methylation profiles associated with litter size, we assayed genomic DNA from the placental tissues of the Berkshire pigs using bisulfite sequencing. Placental tissues were extracted from three pigs in each group (SLG and LLG) and pooled. In total 396.69 and 422.83 million raw reads were generated for these two groups, respectively. The mapped SLG and LLG reads covered 72.26% and 70.55% of the pig genome, respectively (Table 2).

**Table 2:** Summary of sequencing results and reads alignment.

Group	SLG	LLG
Raw Reads (million)	396.69	422.83
Read depth (X)	13.75	14.66
Mapped Reads (million)	286.65	298.31
Mapped Reads (%)	72.26	70.55
Uniquely mapped Reads (million)	260.13	269.77
Uniquely mapped Reads (%)	65.56	63.80

Cytosine methylation occurs in three sequence contexts: CG, CHG, and CHH (where H = A, C, or T). We observed overall genome-wide levels of 82.96% CG, 2.47% CHG, and 2.28% CHH methylation in SLG and 81.60% CG, 2.76% CHG, and 2.51% CHH methylation in LLG (Table 3). The rate of methylated CG was lower in LLG than in SLG. However, both groups had similar genome-wide methylation rates and the rate of methylated CG was much higher than that of CHG and CHH. Therefore, the methylation of CHG and CHH was not considered in further analyses.

**Table 3:** Genome-wide methylation levels in the two groups.

Group	SLG	LLG
No. mCG (%^†^)	19,270,603 (82.96)	18,955,910 (81.60)
No. mCHG* (%)	2,167,423 (2.47)	2,405,935 (2.76)
No. mCHH* (%)	6,557,750 (2.28)	7,163,013 (2.51)

* H = A, C or T

^†^ Number of mCGs / number of CGs in alignment

**Fig 1:**
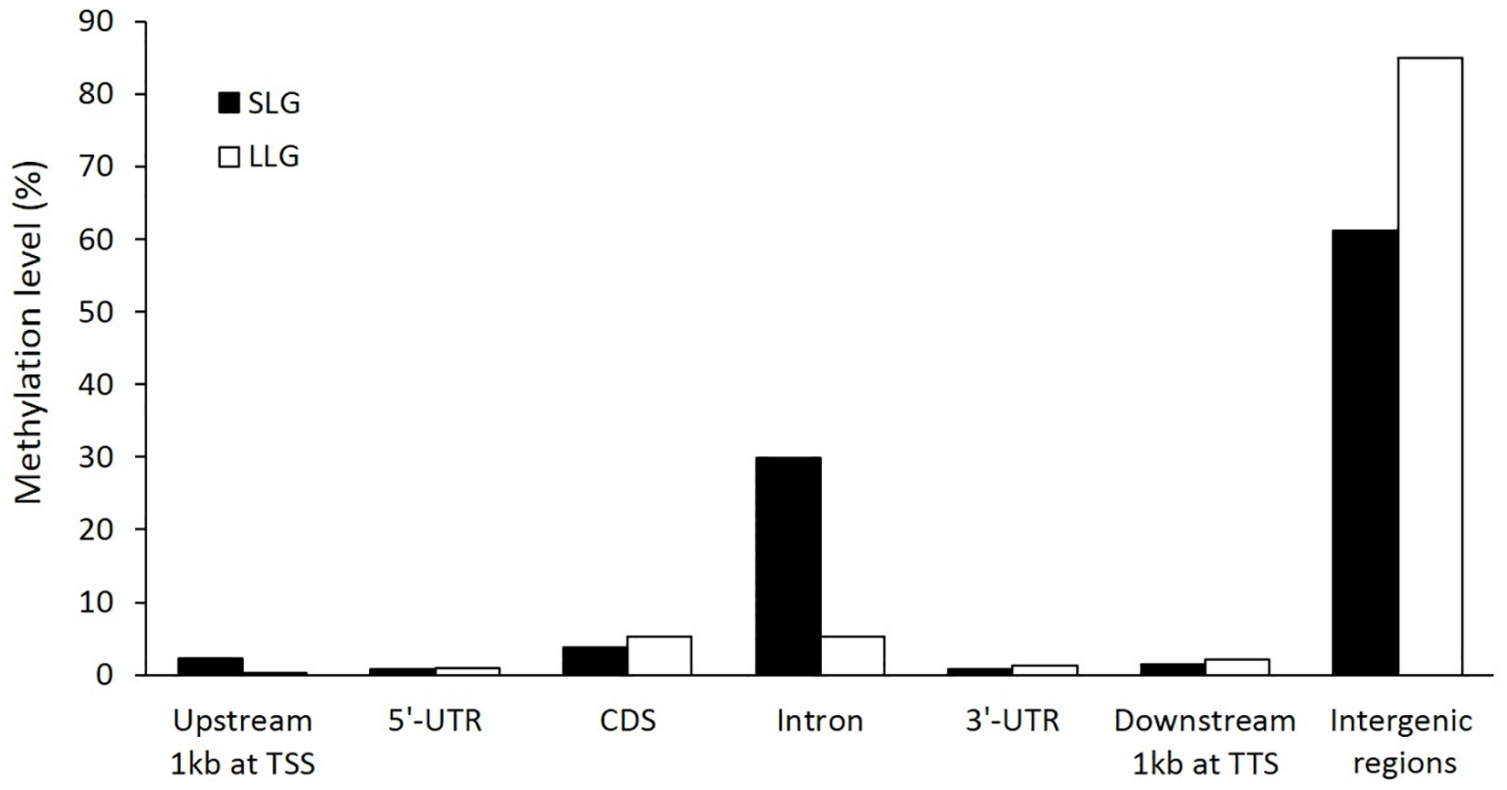
Relative methylation level of mCG for different genomic regions in the two groups. The different genomic regions are shown in the x-axis, and the y-axis shows the methylation level (%).

Next, we analyzed the profiles of CG methylation from various genomic regions, such as the upstream 1 kb at the transcription start site (TSS), 5'-UTR, coding sequence (CDS), introns, 3'-UTR, downstream 1 kb at the transcription termination site (TTS), and other (intergenic) regions, in each group (Fig 1). The methylation levels of the intergenic regions were markedly high in both groups. Intergenic regions with high methylation level mostly correspond to CpG islands and enhancers. Excluding this region, methylation was notably high in CDS and introns with minor methylation in promoter regions (upstream 1 kb at TSS in the sense transcript). The methylation rate in introns was markedly lower in LLG than in SLG.

**Fig 2:**
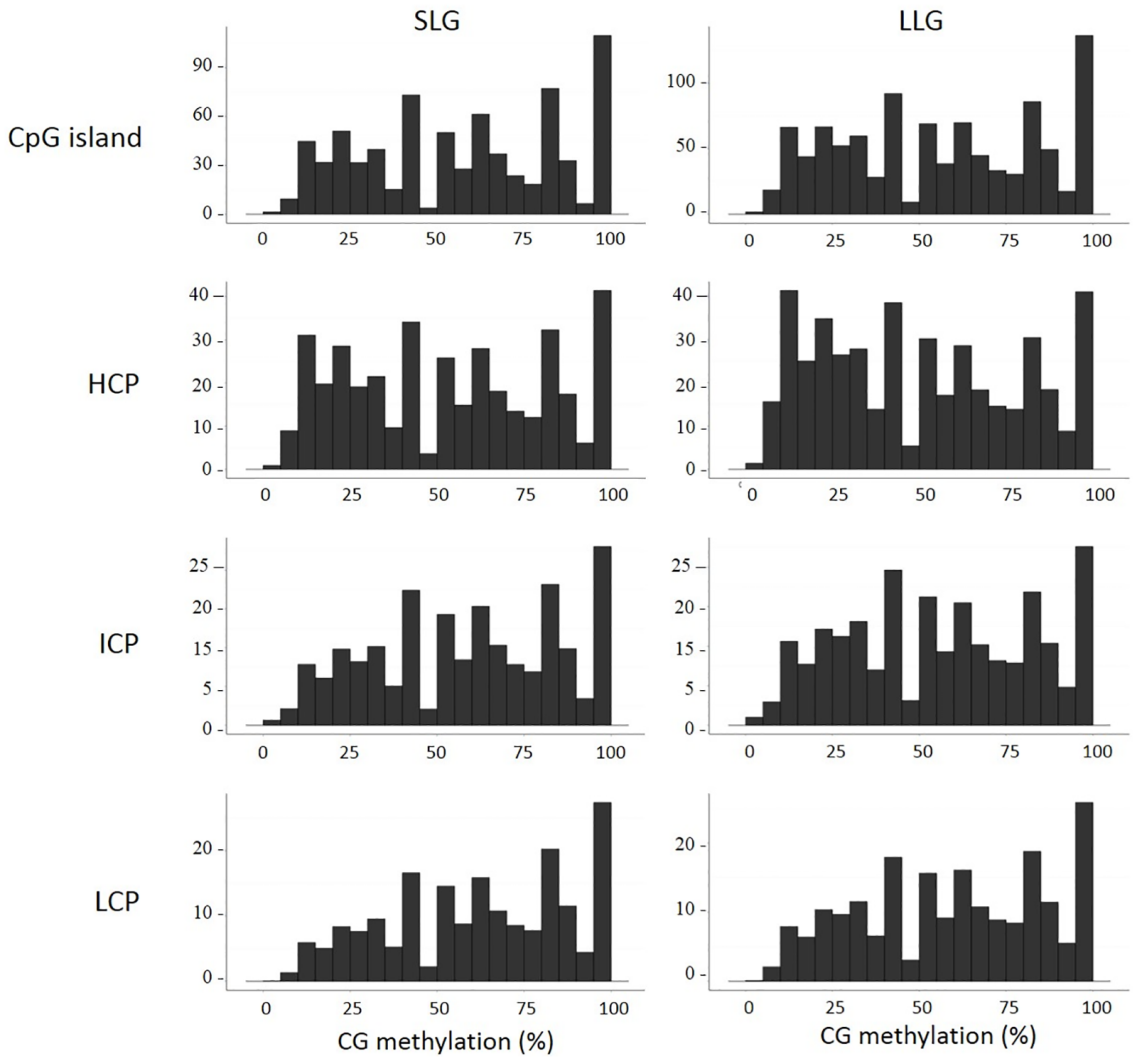
Distribution of CpG methylation levels in CpG island, HCP, ICP, and LCP of each group. The percent methylation for each CpG site is shown in the x-axis, and the y-axis shows the number of CpG sites (x10^2^).

The relationship between DNA methylation in the promoter and gene activity is known to depend on CpG content [21] and we divided the promoters into three categories according to their CpG contents and GC ratios as defined previously [21]. High-CpG promoters (HCP) contain a 500 bp region with a CpG ratio > 0.75 and GC content > 55%. Low-CpG promoters (LCP) do not contain a 500-bp region with a CpG ratio > 0.48. Intermediate-CpG promoters (ICPs) are neither HCP nor LCP. As shown in Fig 2, CpG methylation level in less than the < 50% range was higher in LLG than in SLG.

### Analysis of DMRs and gene ontology

**Fig 3:**
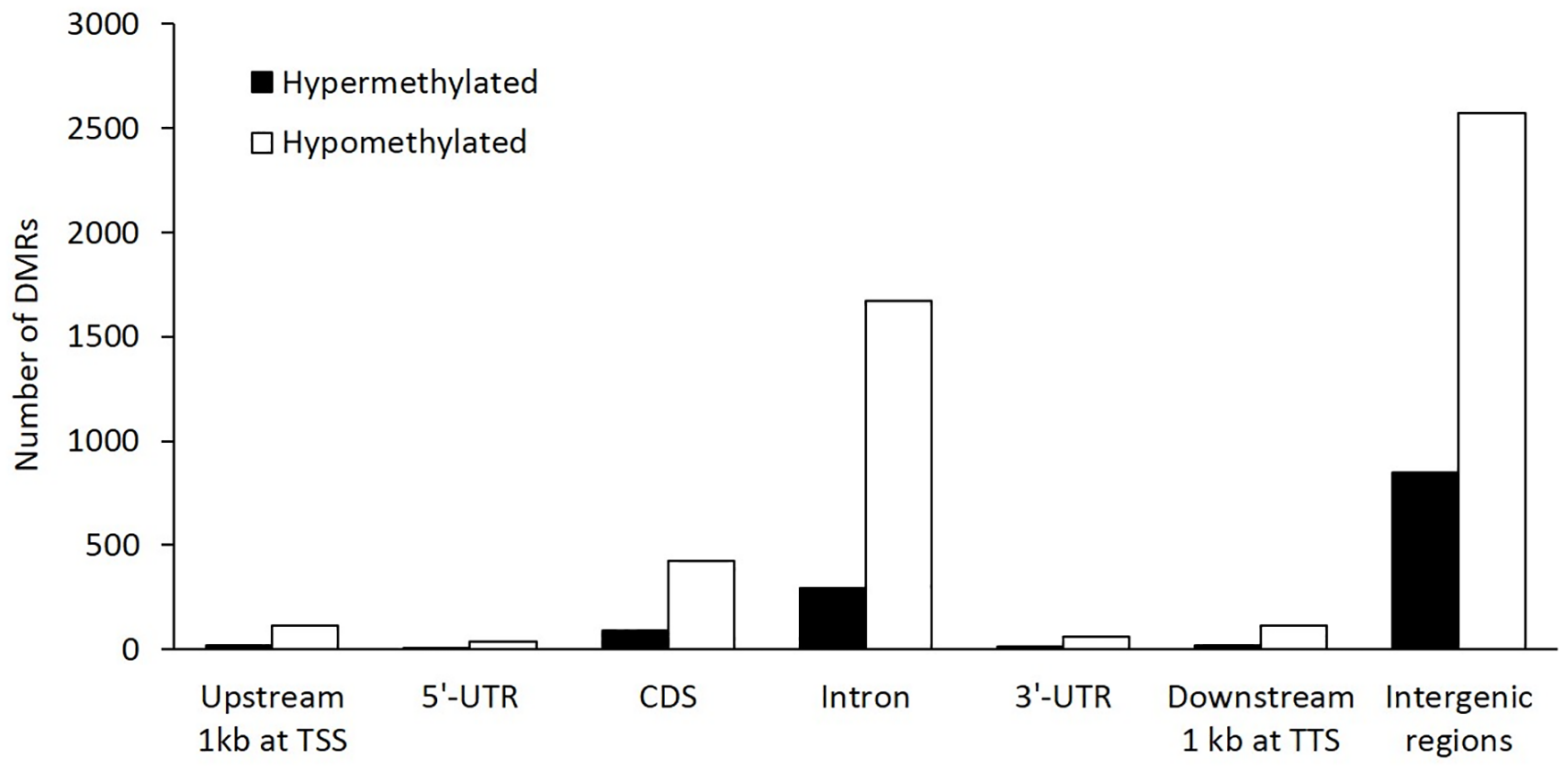
Distribution of DMRs for different genomic regions in LLG compared to SLG. The different genomic regions are shown in the x-axis, and the y-axis shows the number of DMRs.

**Fig 4:**
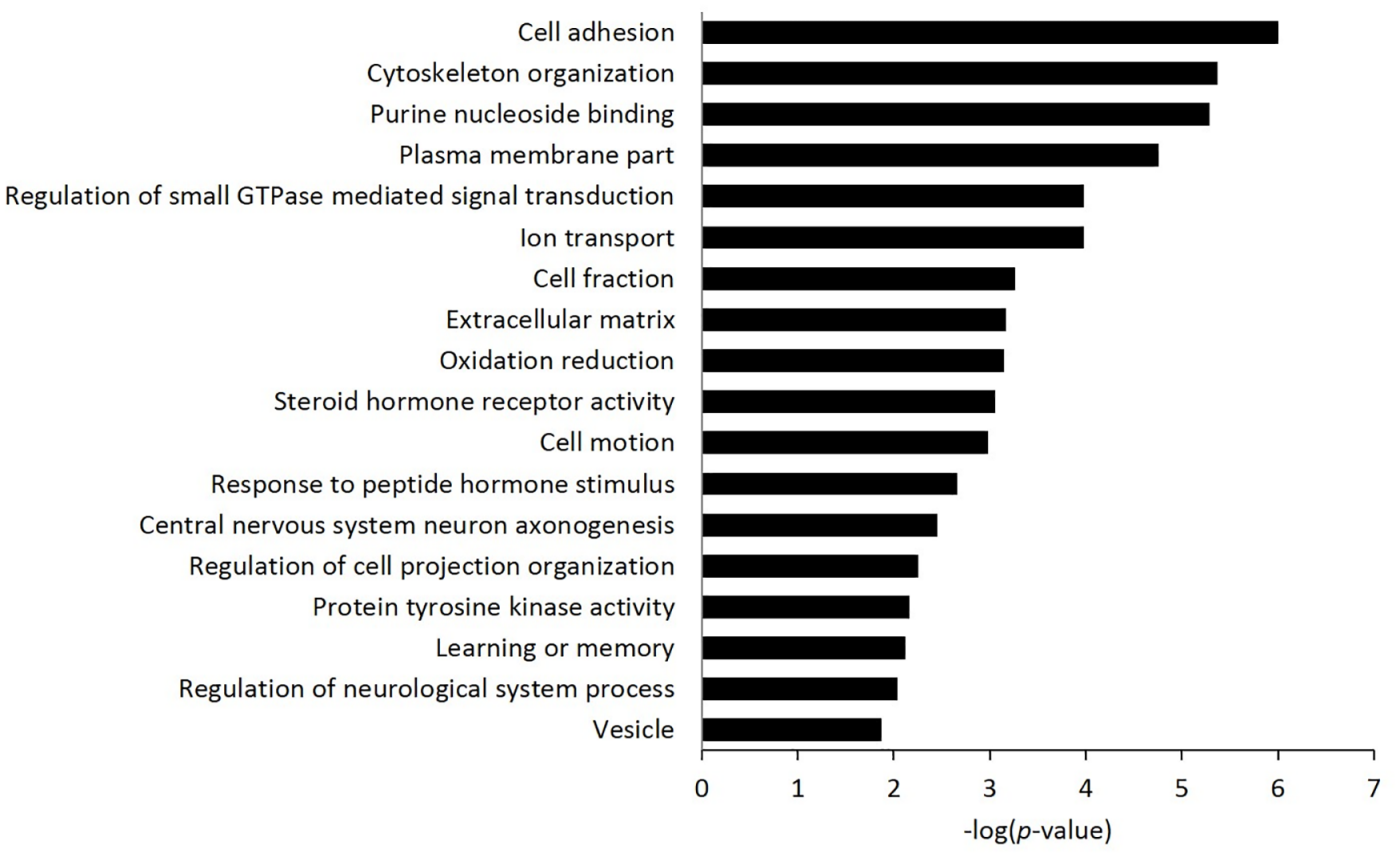
Gene ontology enrichment analysis of the differentially methylated genes. The statistical significance of the enrichment (-log(*p*-value)) in overexpressed genes are shown in the x-axis, and the y-axis shows the different biological processes.

To identify DMRs, the methylation levels of CGs between SLG and LLG were compared and the DMRs of LLGs, which were hypermethylated and hypomethylated with respect to SLG, were examined in different genomic regions: upstream 1 kb at TSS, 5'-UTR, CDS, intron, 3'-UTR, downstream 1 kb at TTS, and intergenic regions. In LLG, 5851 DMRs were discovered: 850 were hypermethylated and 5001 were hypomethylated (S1 File). The majority of these were found in the CDS, intron, and intergenic regions (Fig 3). To investigate the possible related functions of DMRs between SLG and LLG, GO enrichment analysis was performed. The majority of functions were strongly related to cell adhesion, cytoskeleton organization, purine nucleoside binding, and the plasma membrane (Fig 4).

### DEGs positioned in DMRs between the two groups

**Fig 5:**
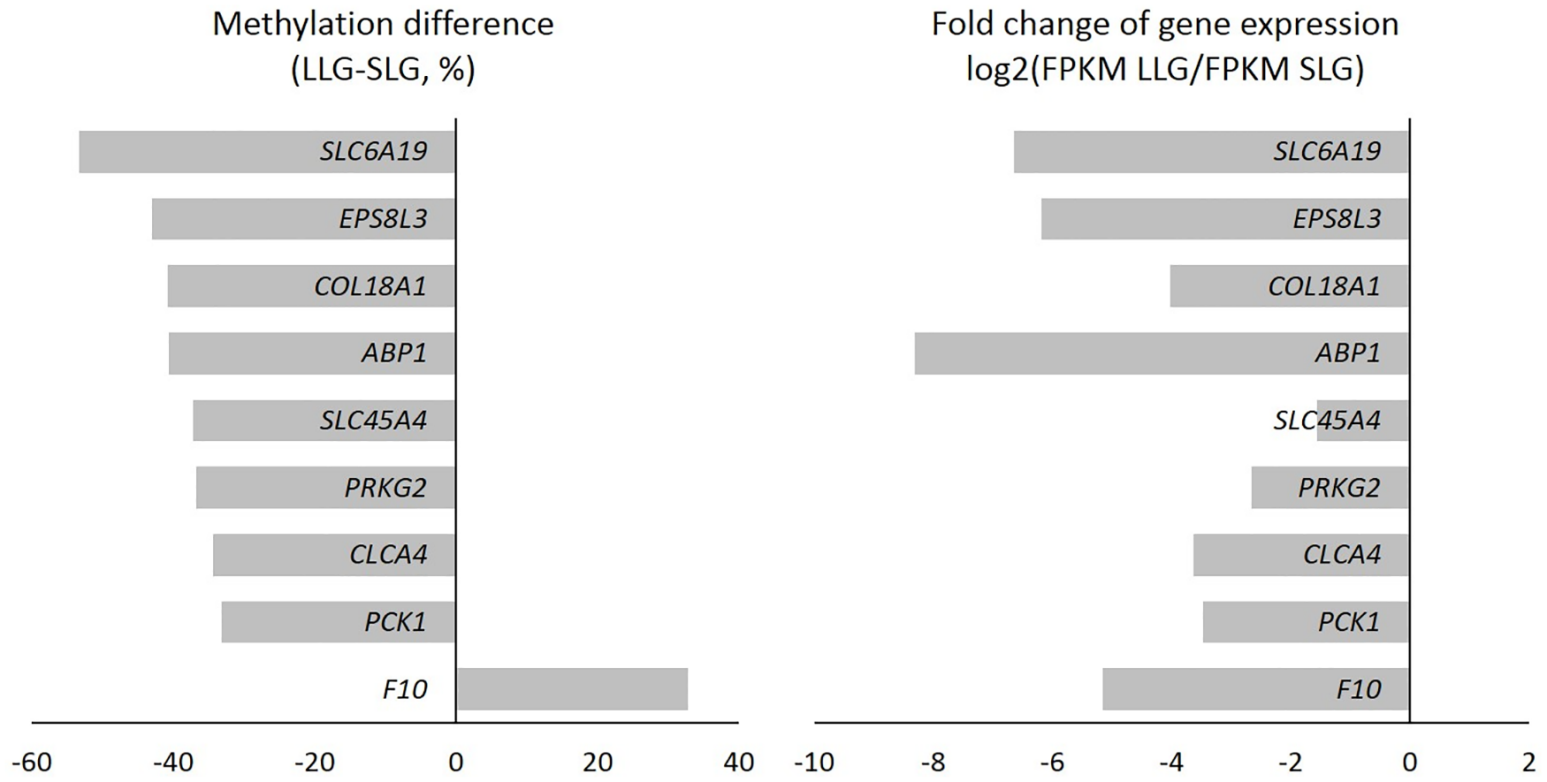
Potential DEGs significantly positively related to DMRs.

Next, RNA-Seq analysis was performed to identify DEGs positioned in DMRs in the two groups. In total, 1591 DEGs were found between SLG and LLG. Of these, 567 and 1024 genes were expressed more in SLG and LLG, respectively. Among 281 DEGs related to fecundity, 37 were upregulated and 241 were downregulated in LLG (S1 and S2 Tables). To analyze DEGs positioned in DMRs, we first identified DMGs that had a difference of more than 30% DNA methylation between the two groups; from among these, we then selected DEGs considering a *p*-value < 0.01 and *q-*value < 0.05 of RNA-Seq. Nine potential DEGs were strongly related to methylation: *SLC6A19*, *EPS8L3*, *COL18A1*, *ABP1*, *SLC45A4*, *PRKG2*, *CLCA4*, *PCK1*, and *F10* (Table 4). Table 5 lists detailed methylation information for these nine genes. All nine genes were methylated in the gene body and positive relationships were observed between the differential expression and differential methylation, except for *F10*. For *F10*, a negative relationship was observed between gene expression and methylation, as shown in Fig 5.

**Table 4:** List of DEGs positioned in DMRs by the litter size.

Gene	Expression value in SLG	Expression value in LLG	Log_2_LLG/SLG (Fold change)	*p*-value	*q*-value	Methylation difference (%, LLG-SLG)
*SLC6A19*	100.5	1	-6.64	5.00×10^−7^	1.61×10^−4^	-53.3
*EPS8L3*	73.6	1	-6.19	1.81×10^−5^	4.09×10^−3^	-43.0
*COL18A1*	2037.8	126	-4.02	4.49×10^−17^	7.1×10^−14^	-40.7
*ABP1*	318.4	1	-8.31	9.97×10^−17^	1.43×10^−13^	-40.6
*SLC45A4*	1570.1	529.7	-1.57	2.56×10^−4^	3.66×10^−2^	-37.2
*PRKG2*	234.8	37.2	-2.66	9.52×10^−5^	1.65×10^−2^	-36.8
*CLCA4*	162.2	13.1	-3.63	1.95×10^−5^	4.35×10^−3^	-34.3
*PCK1*	156.2	14.1	-3.47	4.47×10^−5^	8.51×10^−3^	-33.1
*F10*	71.6	2.01	-5.16	3.03×10^−4^	4.20×10^−2^	32.8

**Table 5:** Methylation region of potential DEGs positioned in DMRs.

Gene	Up-stream 1kb at TSS	5'-UTR	Up-stream 1kb at gene body	Gene body		3'-UTR	Down-stream 1kb at TTS
				CDS	Intron		
*SLC6A19*	-	-	+	-	+	-	-
*EPS8L3*	-	-	+	-	+	-	-
*COL18A1*	-	-	+	-	+	-	-
*ABP1*	+	-	+	+	-	-	-
*SLC45A4*	-	-	+	-	+	-	-
*PRKG2*	-	-	+	-	+	-	-
*CLCA4*	-	-	+	+	-	-	-
*PCK1*	-	-	+	+	+	-	-
*F10*	-	-	+	-	+	-	-

### Verification by RT-qPCR

**Fig 6:**
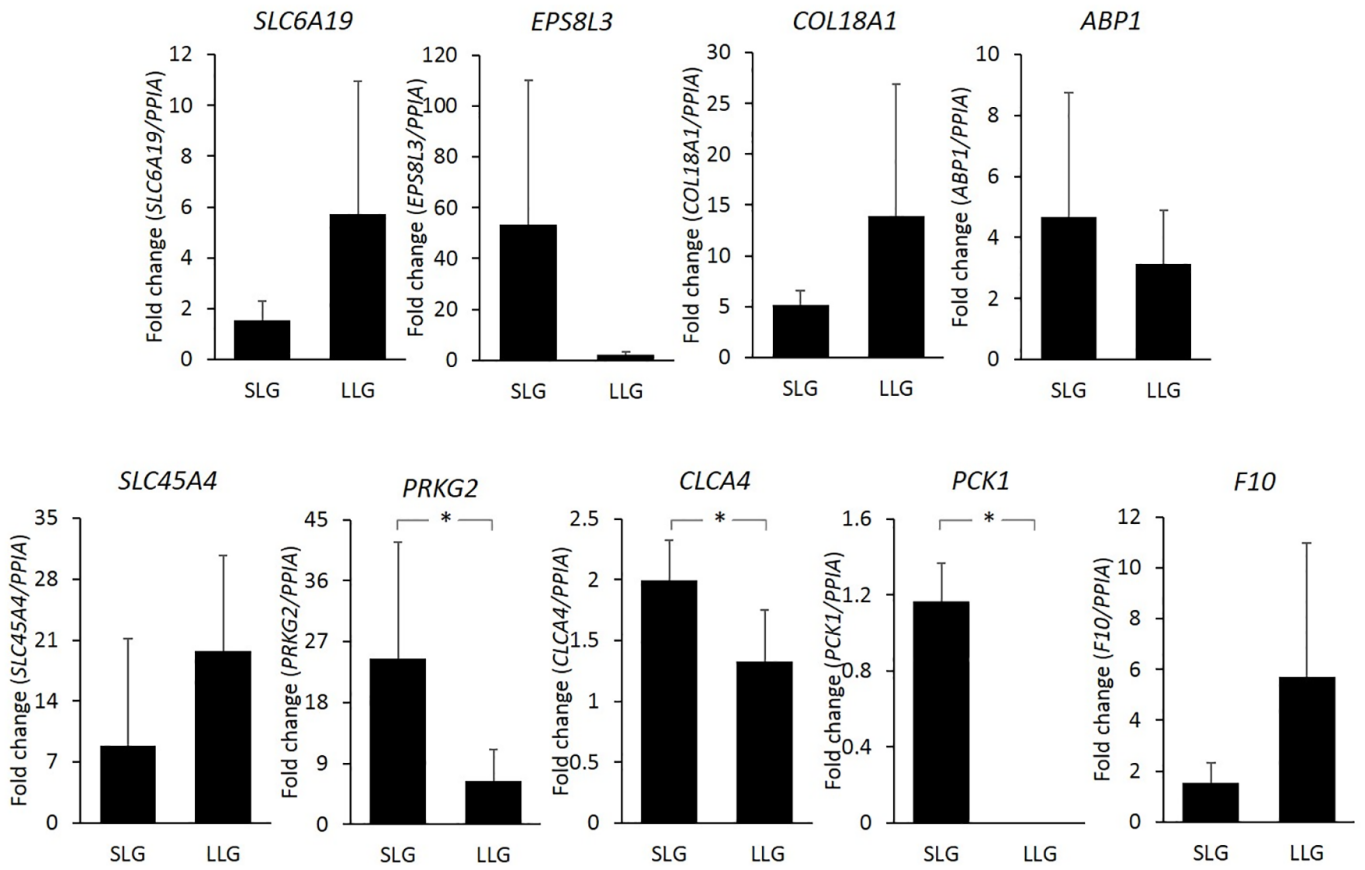
Validation of DEGs by RT-qPCR. The y-axis shows the fold change of gene expression. * Significant difference at *p*<0.05 levels compared between the two groups.

To validate the expression of the nine potential DEGs, RT-qPCR was performed using the same placentas used for RNA-Seq. Fig 6 presents the results. The expression of *PRKG2*, *CLCA4*, and *PCK1* were significantly lower in LLG than in SLG, as with the RNA-Seq results. *PRKG2* and *CLCA4* mRNA levels were 3.8- and 1.5-fold lower in LLG, respectively. *PCK1* expression was minimal in LLG and strong in SLG. These genes were therefore identified as candidate genetic markers for litter size in Berkshire pigs.

### PCR-based methylation analysis of three genes in blood samples

**Fig 7:**
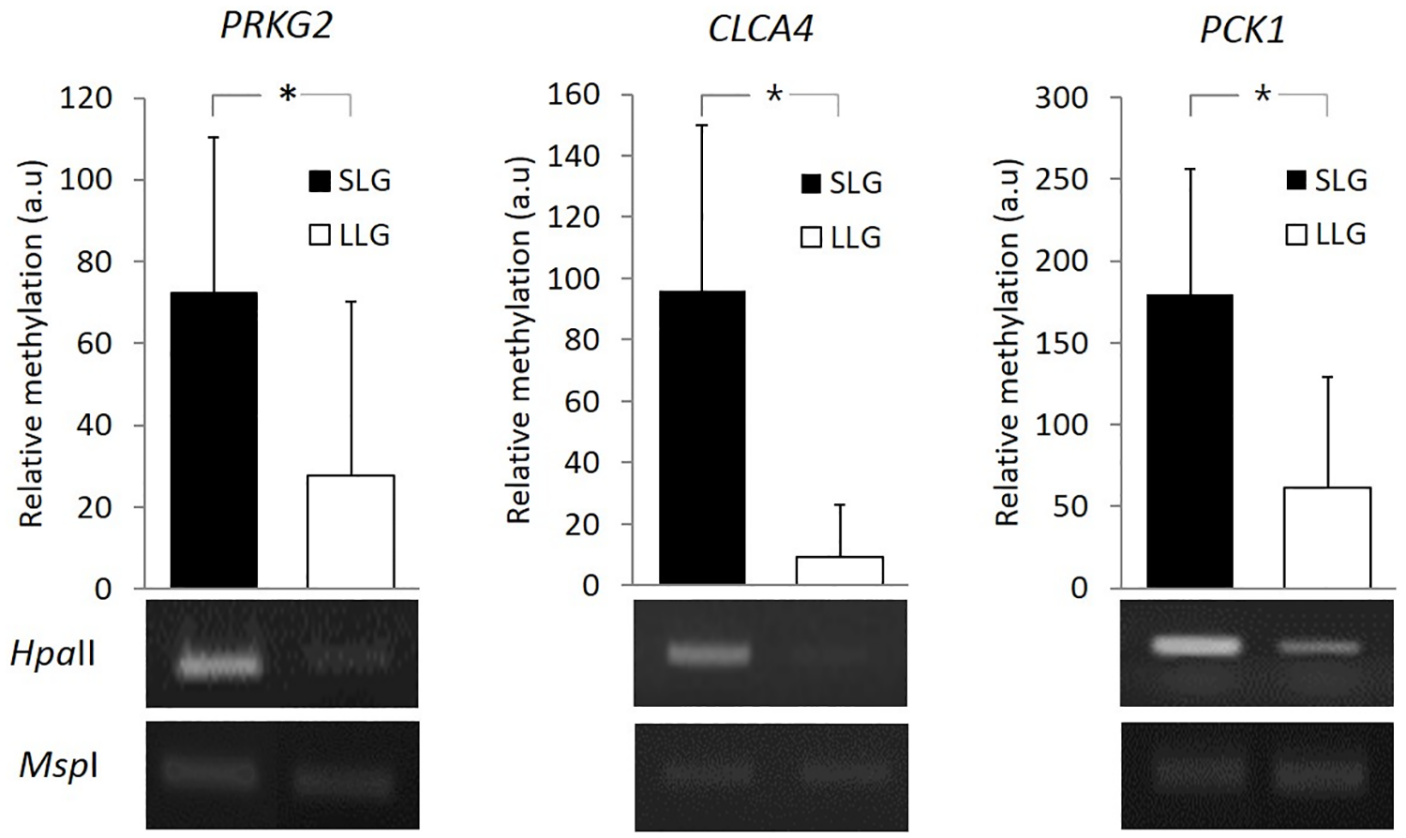
Analysis of *PRKG2*, *CLCA4*, and *PCK1* genes methylation in blood by PCR-based methylation assay. The y-axis shows the relative methylation levels of genes. The band shows DNA digested with *Hpa*II or *Msp*I. * Significant difference at *p*<0.05 levels compared between the two groups.

To assess the diagnostic capability of the three candidate genes as blood-based biomarkers, we investigated the methylation levels of these genes using PCR-based methylation assay. This assay allows very rapid screening of the methylation status of a genomic DNA region, digested with the methylation-sensitive restriction enzyme *Hpa*II and its methylation-insensitive isoschizomer, *Msp*I. As shown in Fig 7, we confirmed that *PRKG2*, *CLCA4*, and *PCK1* were relatively hypomethylated in LLG, consistent with the results of bisulfite sequencing analysis.

## Discussion

The placenta is an important endocrine organ throughout pregnancy, and markedly affects fetal health by supplying nutrients [22]. Recently, Lee *et al*. [23] found prolificacy-related proteins such as PSA and RBP4 in porcine placenta using proteomic analyses. These proteins were overexpressed in LLG, and the authors suggested that their expression plays a crucial role in placental efficiency. Another study compared the expression and genome-wide DNA methylation patterns of 18 imprinted genes between mid-gestational, aborted, cloned porcine fetuses and placentas [24]. However, no studies have investigated methylation in placental tissue in relation to litter size in pigs. Moreover, the DNA methylation status of the promoter and gene body regions can affect gene expression via changes in chromatin structure or transcription efficiency [25, 26]. The present study is the first to systematically compare the genome-wide DNA methylation profiles of placental tissue from Berkshire pigs based on litter size and identify DEGs positioned in DMRs.

Over 80% of all CG sites appeared to be methylated in both groups. This was in contrast with CHG and, especially, CHH methylation was found at much lower levels. The methylation levels of the gene body regions were much higher than the promoter regions and 5'- and 3'-UTR in SLG and LLG. Hypermethylation or hypomethylation of the gene body regions in the pig genome is known to regulate gene expression [27, 28]. Most of the genes had one or more methylated regions within their promoter and gene body regions. Additionally, considerably more hypomethylated than hypermethylated regions were observed. DNA hypomethylation affects genes that alter cell growth and development, histone remodeling, apoptosis, and cellular proliferation [29, 30]. These observations suggest that the hypomethylation of a number of reproduction-associated genes in LLGs, resulting in a larger litter size.

Epigenetic regulators, such as DNA methylation and histone modification have essential functions in placental growth and development. GO enrichment analyses were performed to investigate the potential functions of DMGs responsible for phenotype differences in biological processes. We identified several important biological processes in placenta, such as cell adhesion, cytoskeleton organization, purine nucleoside binding, and plasma membrane activity.

Next, we assessed DEGs positioned in DMRs and found nine hypomethylated genes with decreased gene expression in LLG compared to SLG using RNA-Seq. The role of methylation in gene expression has been studied widely, particularly in cancer research. Gene silencing mediated by aberrant promoter DNA hypermethylation is one of the key features of cancer, which involves a negative correlation between DNA methylation and gene expression. While DNA methylation in gene promoters is strongly associated with gene silencing [21, 31, 32], methylation in gene bodies is mostly associated with transcription elongation [27, 33, 34] and has been speculated to have a functional role in this context [35]. Recent studies have described different correlations between transcription and intragenic DNA methylation [36, 37]. Overall, gene body methylation is common in humans [38] and is conserved across plants and animals [39, 40], and methylation of the gene body is positively correlated with gene expression [38, 41, 42]. Consistent with the reported positive correlations, we observed that methylation in the gene body in eight of nine genes was positively related to their gene expression. The RNA-Seq results were confirmed by RT-qPCR, and three genes (*PRKG2*, *CLCA4*, and *PCK1*) yielded the same results. Furthermore, these three genes were verified as potential blood-based epigenetic markers by PCR-based methylation analysis.

Protein kinase, cGMP-dependent type II (*PRKG2*) encodes a serine/threonine-specific protein kinase that is activated by cGMP. In mammals, it phosphorylates several biologically important targets and is implicated in the regulation of smooth muscle relaxation, platelet function, sperm metabolism, cell division, and nucleic acid synthesis [43]. The chorionic villi of the placenta increase the surface area for absorption, similar to the intestinal villi. As it has been characterized as a dimer in the intestinal brush border membrane [44], porcine *PRKG2* can be assumed to influence litter size via the placenta.

Chloride channel, calcium activated, family member 4 (*CLCA4*) encodes a calcium-sensitive chloride conductance protein. The family of calcium-activated chloride channels (CaCCs) is a heterogeneous group of ligand-gated ion channels for chloride that has been identified in many epithelial and endothelial cell types, as well as in smooth muscle cells [45]. CaCCs have been studied extensively in smooth muscle cells from a variety of tissues, including the portal vein, trachea, lymphatic vessels, and pulmonary artery in mammals [46–49]. The physiological function of endogenous CaCCs in smooth muscle cells is to cause membrane depolarization and sustain contraction. Moreover, the placenta villi are composed of three layers with different cell types, including vascular smooth muscle cells [50]. CaCCs such as *CLCA4* can alter placental function by affecting the smooth muscle cells and ultimately influence litter size in pigs.

Phosphoenolpyruvate carboxykinase 1 (PCK1) encodes an enzyme that regulates gluconeogenesis. Overexpression of this enzyme results in symptoms of type II diabetes, by far the most common form of diabetes in humans. Due to the importance of blood glucose homeostasis, several hormones regulate a set of genes (including PCK1) in the liver that modulate the rate of glucose synthesis [51]. The activity of phosphoenolpyruvate carboxylase (PEPCK), which exists in cytosolic (PCK1) and mitochondrial (PCK2) forms, has been detected in the human placenta, suggesting that gluconeogenesis can occur in the placenta. A decrease in the activity of this enzyme in placenta is associated with placental growth during the course of normal gestation [52, 53]. Several studies have reported increased fetal plasma cortisol and an associated increase in gluconeogenesis in response to a reduction of maternal nutrients [54, 55]. Changes in PEPCK in response to decreased fetal nutrient availability have been extensively investigated in rodents and sheep [55, 56]. We postulate that PCK1 affects litter size by mediating maternal–fetal nutrient transport in the placenta in pregnancy.

In conclusion, we investigated genome-wide DNA methylation and gene expression in two groups of pigs based on litter size using genome-wide bisulfite sequencing and RNA-Seq. The analysis of differential DNA methylation and differential gene expression detected three genes that were strongly associated with litter size. While many studies have addressed the importance of gene selection in animal reproduction, few have done this considering DMRs. Our study suggests that epigenetic analysis might be a novel tool for selecting higher fecundity in individual pigs. Compared to using gene expression properties, DMRs can easily be determined from blood samples taken from individual pigs. It has been reported that even when individuals of the same species are raised in the same environment, a number of factors can still influence reproduction. Considering these complicated influential factors, our approach might be useful to check the fecundity of individual pigs. The results suggest that the *PRKG2*, *CLCA4*, and *PCK1* genes can be used as juvenile selection markers for hyperprolific sows. Although the pooled sampling method used in this study, which may mask some variation, is not the best approach for genome-wide analysis, it is cost-effective. Its weaknesses could be improved by further validation. Further studies with larger populations are needed to confirm the usability of these genes as stable epigenetic markers.

## Supporting information

S1 Fig The average methylation levels in the two goups. (A) Genes; (B) Transposable elements (TE); (C) Different genic rigions.
(TIF)

S1 File List of DMRs between SLG and LLG. (XLSX)

S1 Table List of upregulated DEGs related to fecundity in LLG. (DOCX)

S2 Table List of downregulated DEGs related to fecundity in LLG. (DOCX)
