# Supplementary figures and images for: DNA methylation patterns and gene expression associated with litter size in Berkshire pig placenta
Source: PLoS One. 2017 Sep 7;12(9):e0184539. doi: 10.1371/journal.pone.0184539 (PMC5589248; doi:10.1371/journal.pone.0184539)

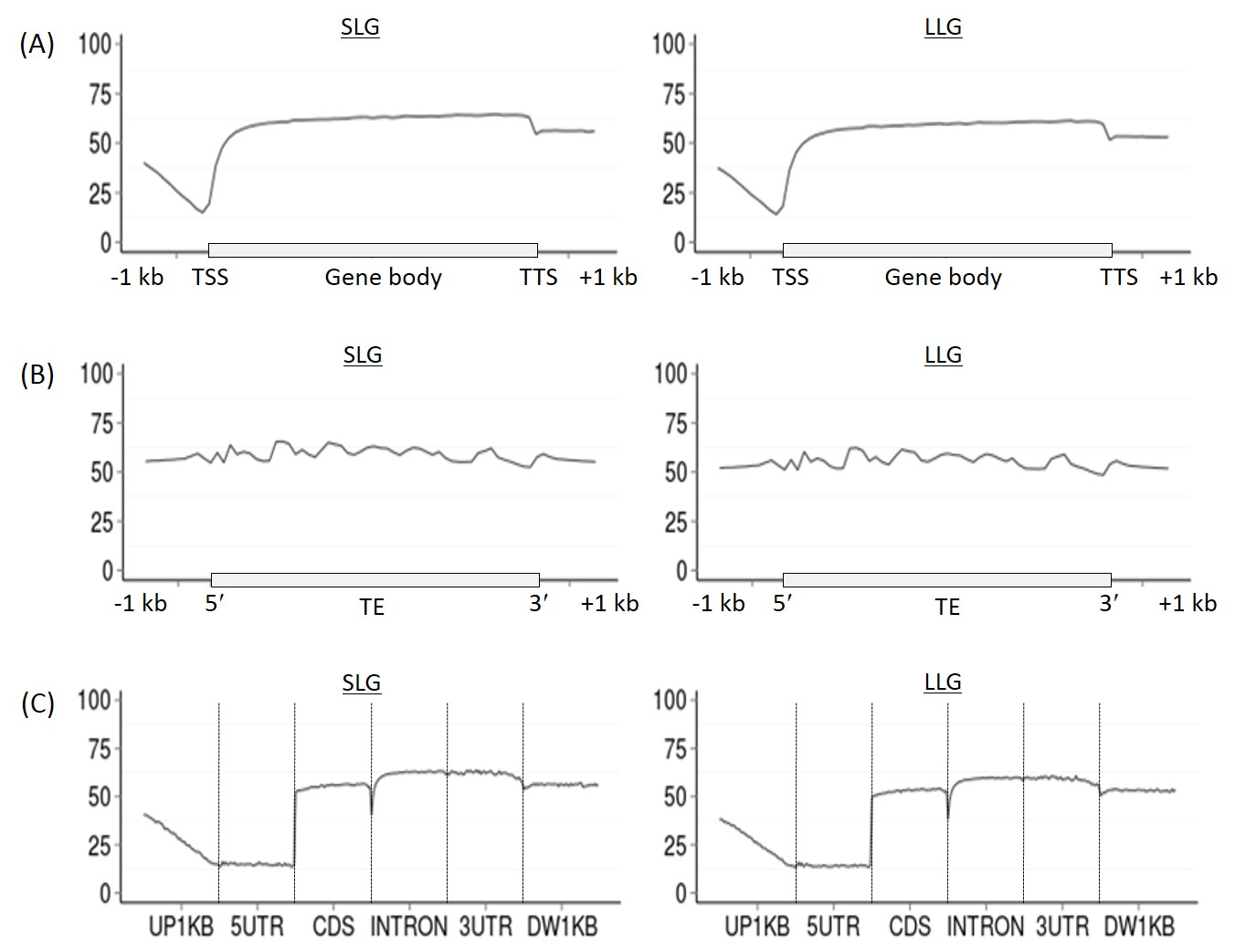

Supplement: S1 Fig — (A) Genes; (B) Transposable elements (TE); (C) Different genic rigions. (TIF) [file pone.0184539.s001.tif]
