# Supplementary material for: DNA methylation patterns and gene expression associated with litter size in Berkshire pig placenta
Source: PLoS One. 2017 Sep 7;12(9):e0184539. doi: 10.1371/journal.pone.0184539 (PMC5589248; doi:10.1371/journal.pone.0184539)
Supplement: S1 Table — (DOCX) [file pone.0184539.s003.docx]

**S1 Table. List of upregulated DEGs related to fecundity in LLG.**

| No. | Accession no. | Name | Chr | SLG | LLG | log2(LLG/SLG) | p-value | q-value |
| --- | --- | --- | --- | --- | --- | --- | --- | --- |
| 1 | ENSSSCG00000023987 | - | 2 | 154.23 | 1628.16 | 3.4 | 0 | 0 |
| 2 | ENSSSCG00000016900 | - | 16 | 894.51 | 2994.01 | 1.74 | 2.42E-05 | 0.005243 |
| 3 | ENSSSCG00000014725 | HBB | 9 | 21151.9 | 65691.4 | 1.63 | 2.67E-05 | 0.005596 |
| 4 | ENSSSCG00000010312 | PLAU | 14 | 971.13 | 3032.21 | 1.64 | 6.39E-05 | 0.011612 |
| 5 | ENSSSCG00000021865 | INHBA | 18 | 35.82 | 236.18 | 2.72 | 6.84E-05 | 0.012154 |
| 6 | ENSSSCG00000012347 | ALAS2 | X | 1043.77 | 3127.68 | 1.58 | 0.000109 | 0.018402 |
| 7 | ENSSSCG00000029488 | ADAMTS1 | 13 | 42.78 | 245.23 | 2.52 | 0.000146 | 0.023577 |
| 8 | ENSSSCG00000002623 | GSTA2 | 7 | 79.6 | 360.81 | 2.18 | 0.00015 | 0.023992 |
| 9 | ENSSSCG00000010224 | EGR2 | 14 | 419.89 | 1294.49 | 1.62 | 0.000216 | 0.032185 |
| 10 | ENSSSCG00000018086 | ND4L | MT | 1964.15 | 5440.28 | 1.47 | 0.000227 | 0.033423 |
| 11 | ENSSSCG00000013784 | DNAJB1 | 2 | 7182.98 | 18692.7 | 1.38 | 0.00039 | 0.052332 |
| 12 | ENSSSCG00000011951 | NFKBIZ | 13 | 125.37 | 452.27 | 1.85 | 0.000482 | 0.062452 |
| 13 | ENSSSCG00000004509 | LIPG | 1 | 380.09 | 1082.43 | 1.51 | 0.000704 | 0.083152 |
| 14 | ENSSSCG00000017698 | CCL4 | 12 | 6.96 | 90.45 | 3.7 | 0.000746 | 0.086822 |
| 15 | ENSSSCG00000023279 | SH3TC2 | 2 | 13.93 | 117.59 | 3.08 | 0.000819 | 0.092984 |
| 16 | ENSSSCG00000028322 | BTG2 | 9 | 6824.77 | 16474.6 | 1.27 | 0.001063 | 0.116301 |
| 17 | ENSSSCG00000011877 | CD86 | 13 | 347.26 | 966.85 | 1.48 | 0.001066 | 0.116301 |
| 18 | ENSSSCG00000003065 | PLAUR | 6 | 595.02 | 1562.84 | 1.39 | 0.001078 | 0.11682 |
| 19 | ENSSSCG00000015334 | PDK4 | 9 | 6502.39 | 15538.9 | 1.26 | 0.001216 | 0.130291 |
| 20 | ENSSSCG00000003451 | PDPN | 6 | 212.93 | 623.12 | 1.55 | 0.001435 | 0.150372 |
| 21 | ENSSSCG00000015476 | CHI3L1 | 9 | 1458.68 | 3499.55 | 1.26 | 0.001705 | 0.168598 |
| 22 | ENSSSCG00000007554 | ZFAND2A | 3 | 185.07 | 537.7 | 1.54 | 0.002075 | 0.194241 |
| 23 | ENSSSCG00000007032 | PLAT | 17 | 2211.91 | 5009.12 | 1.18 | 0.002909 | 0.248255 |
| 24 | ENSSSCG00000007978 | HBA | 3 | 6759.1 | 14894.7 | 1.14 | 0.003268 | 0.272125 |
| 25 | ENSSSCG00000006595 | IVL | 4 | 1531.32 | 3460.35 | 1.18 | 0.003396 | 0.281262 |
| 26 | ENSSSCG00000009482 | SPRY2 | 11 | 250.74 | 646.24 | 1.37 | 0.004216 | 0.331832 |
| 27 | ENSSSCG00000013408 | ADM | 2 | 847.75 | 1923.65 | 1.18 | 0.004421 | 0.342885 |
| 28 | ENSSSCG00000015595 | ATF3 | 9 | 2873.59 | 6166.93 | 1.1 | 0.005055 | 0.365167 |
| 29 | ENSSSCG00000008953 | IL8 | 8 | 23.88 | 121.61 | 2.35 | 0.005452 | 0.388535 |
| 30 | ENSSSCG00000013067 | PHEROC | 2 | 18.91 | 107.54 | 2.51 | 0.005504 | 0.390473 |
| 31 | ENSSSCG00000004261 | - | 1 | 196.02 | 507.55 | 1.37 | 0.006047 | 0.418313 |
| 32 | ENSSSCG00000017723 | CCL2 | 12 | 135.32 | 374.88 | 1.47 | 0.006398 | 0.430809 |
| 33 | ENSSSCG00000003766 | DNAJB4 | 6 | 1054.71 | 2269.38 | 1.11 | 0.006946 | 0.450294 |
| 34 | ENSSSCG00000011265 | XIRP1 | 13 | 19.9 | 106.53 | 2.42 | 0.00711 | 0.451729 |
| 35 | ENSSSCG00000011208 | ZNF385D | 13 | 452.73 | 1025.14 | 1.18 | 0.00753 | 0.472748 |
| 36 | ENSSSCG00000006070 | COX6C | 4 | 1339.28 | 2827.18 | 1.08 | 0.007639 | 0.477697 |
| 37 | ENSSSCG00000003155 | PPP1R15A | 6 | 2548.22 | 5244.3 | 1.04 | 0.00823 | 0.50079 |
