# Supplementary material for: DNA methylation patterns and gene expression associated with litter size in Berkshire pig placenta
Source: PLoS One. 2017 Sep 7;12(9):e0184539. doi: 10.1371/journal.pone.0184539 (PMC5589248; doi:10.1371/journal.pone.0184539)
Supplement: S2 Table — (DOCX) [file pone.0184539.s004.docx]

**S2 Table. List of downregulated DEGs related to fecundity in LLG.**

| No | Accession | Name | Chr | SLG | LLG | log2 (SLG/LLG) | p-value | q-value |
| --- | --- | --- | --- | --- | --- | --- | --- | --- |
| 1 | ENSSSCG00000000251 | KRT1 | 5 | 2441.75 | 2.01 | -10.2 | 0 | 0 |
| 2 | ENSSSCG00000000248 | KRT5 | 5 | 3237.76 | 44.22 | -6.19 | 0 | 0 |
| 3 | ENSSSCG00000012178 | EIF2S3 | Y | 901.48 | 0 | -9.81 | 0 | 0 |
| 4 | ENSSSCG00000026430 | DDX3Y | Y | 812.92 | 0 | -9.66 | 0 | 0 |
| 5 | ENSSSCG00000024610 | KRT4 | 5 | 2931.3 | 89.45 | -5.03 | 0 | 0 |
| 6 | ENSSSCG00000028522 | KRT10 | 12 | 1523.36 | 41.21 | -5.21 | 0 | 0 |
| 7 | ENSSSCG00000005394 | ALDOB | 1 | 488.55 | 0 | -8.93 | 0 | 0 |
| 8 | ENSSSCG00000008596 | - | 3 | 421.88 | 0 | -8.71 | 0 | 0 |
| 9 | ENSSSCG00000023945 | - | 13 | 476.61 | 2.01 | -7.89 | 0 | 0 |
| 10 | ENSSSCG00000030160 | COL18A1 | 13 | 2037.78 | 125.63 | -4.02 | 0 | 0 |
| 11 | ENSSSCG00000016442 | ABP1 | 18 | 318.4 | 0 | -8.31 | 0 | 0 |
| 12 | ENSSSCG00000011453 | ITIH4 | 13 | 573.13 | 18.09 | -4.99 | 0 | 0 |
| 13 | ENSSSCG00000002709 | - | 6 | 908.44 | 58.29 | -3.96 | 0 | 0 |
| 14 | ENSSSCG00000017433 | KRT14 | 12 | 944.26 | 66.33 | -3.83 | 0 | 0 |
| 15 | ENSSSCG00000011837 | MFI2 | 13 | 461.69 | 17.09 | -4.76 | 0 | 0 |
| 16 | ENSSSCG00000024816 | CEBPB | 17 | 264.67 | 2.01 | -7.04 | 0 | 0 |
| 17 | ENSSSCG00000016159 | CPS1 | 15 | 260.69 | 2.01 | -7.02 | 0 | 0 |
| 18 | ENSSSCG00000001724 | MEP1A | 7 | 213.93 | 0 | -7.73 | 0 | 0 |
| 19 | ENSSSCG00000005832 | MAMDC4 | 1 | 925.36 | 77.39 | -3.58 | 0 | 0 |
| 20 | ENSSSCG00000016247 | TM4SF20 | 15 | 200 | 0 | -7.64 | 0 | 0 |
| 21 | ENSSSCG00000011663 | RBP2 | 13 | 192.04 | 0 | -7.58 | 0 | 0 |
| 22 | ENSSSCG00000012179 | ZFY | Y | 186.07 | 0 | -7.53 | 0 | 0 |
| 23 | ENSSSCG00000015068 | APOA4 | 9 | 216.91 | 3.02 | -6.17 | 0 | 1E-07 |
| 24 | ENSSSCG00000030013 | - | 15 | 190.05 | 2.01 | -6.56 | 0 | 4E-07 |
| 25 | ENSSSCG00000003080 | IGSF23 | 6 | 155.22 | 0 | -7.27 | 0 | 4E-07 |
| 26 | ENSSSCG00000029864 | EIF1AX | Y | 147.26 | 0 | -7.19 | 0 | 9E-07 |
| 27 | ENSSSCG00000006106 | CDH17 | 4 | 227.86 | 6.03 | -5.24 | 0 | 9E-07 |
| 28 | ENSSSCG00000011723 | MME | 13 | 194.03 | 3.02 | -6.01 | 0 | 9E-07 |
| 29 | ENSSSCG00000021728 | LGALS2 | 5 | 145.27 | 0 | -7.18 | 0 | 1.1E-06 |
| 30 | ENSSSCG00000009179 | MTTP | 8 | 279.6 | 12.06 | -4.53 | 0 | 1.1E-06 |
| 31 | ENSSSCG00000013231 | C1QTNF4 | 2 | 14059.5 | 2634.21 | -2.42 | 0 | 1.2E-06 |
| 32 | ENSSSCG00000026547 | SLC45A3 | 9 | 4845.7 | 865.34 | -2.49 | 0 | 1.2E-06 |
| 33 | ENSSSCG00000023949 | PIM3 | 5 | 465.67 | 41.21 | -3.5 | 0 | 0.000002 |
| 34 | ENSSSCG00000007371 | HNF4A | 17 | 131.34 | 0 | -7.03 | 0 | 4.9E-06 |
| 35 | ENSSSCG00000005395 | - | 1 | 148.26 | 1 | -7.2 | 0 | 0.000005 |
| 36 | ENSSSCG00000014633 | PRKCDBP | 9 | 577.11 | 65.33 | -3.14 | 0 | 0.000005 |
| 37 | ENSSSCG00000014055 | CDHR2 | 2 | 201.99 | 6.03 | -5.07 | 0 | 6.2E-06 |
| 38 | ENSSSCG00000023662 | CHST3 | 14 | 6174.04 | 1242.23 | -2.31 | 0 | 6.8E-06 |
| 39 | ENSSSCG00000008595 | APOB | 3 | 122.39 | 0 | -6.93 | 0 | 1.26E-05 |
| 40 | ENSSSCG00000016284 | - | 15 | 119.4 | 0 | -6.89 | 0 | 1.78E-05 |
| 41 | ENSSSCG00000017444 | KRT15 | 12 | 148.26 | 2.01 | -6.2 | 0 | 1.83E-05 |
| 42 | ENSSSCG00000002866 | CEBPA | 6 | 1048.74 | 175.88 | -2.58 | 1E-07 | 2.23E-05 |
| 43 | ENSSSCG00000017445 | KRT13 | 12 | 2625.83 | 547.75 | -2.26 | 1E-07 | 3.92E-05 |
| 44 | ENSSSCG00000016196 | VIL1 | 15 | 218.9 | 12.06 | -4.18 | 2E-07 | 5.83E-05 |
| 45 | ENSSSCG00000010437 | PAPSS2 | 14 | 7256.61 | 1679.42 | -2.11 | 2E-07 | 0.000063 |
| 46 | ENSSSCG00000015249 | ADAMTS8 | 9 | 546.26 | 78.39 | -2.8 | 3E-07 | 8.93E-05 |
| 47 | ENSSSCG00000025858 | ELN | 3 | 4986.99 | 1181.93 | -2.08 | 4E-07 | 0.000125 |
| 48 | ENSSSCG00000017120 | SLC6A19 | 16 | 100.5 | 0 | -6.64 | 5E-07 | 0.000162 |
| 49 | ENSSSCG00000001906 | CYP1A1 | 7 | 385.07 | 48.24 | -3 | 7E-07 | 0.00021 |
| 50 | ENSSSCG00000008294 | ACTG2 | 3 | 4245.71 | 1034.19 | -2.04 | 7E-07 | 0.00022 |
| 51 | ENSSSCG00000007549 | CYP2W1 | 3 | 573.13 | 95.48 | -2.59 | 1.1E-06 | 0.000329 |
| 52 | ENSSSCG00000011277 | CCK | 13 | 179.1 | 10.05 | -4.16 | 1.3E-06 | 0.000398 |
| 53 | ENSSSCG00000020694 | DSG1 | 6 | 165.17 | 8.04 | -4.36 | 1.4E-06 | 0.00042 |
| 54 | ENSSSCG00000010036 | SLC5A1 | 14 | 193.03 | 14.07 | -3.78 | 2.8E-06 | 0.000811 |
| 55 | ENSSSCG00000024092 | KRT77 | 5 | 84.58 | 0 | -6.39 | 4.1E-06 | 0.001151 |
| 56 | ENSSSCG00000028846 | - | 13 | 1637.79 | 410.06 | -2 | 4.8E-06 | 0.00132 |
| 57 | ENSSSCG00000022484 | CH242-228L8.2 | X | 275.62 | 33.17 | -3.05 | 4.9E-06 | 0.001326 |
| 58 | ENSSSCG00000020785 | DES | 15 | 2366.13 | 621.12 | -1.93 | 4.9E-06 | 0.001326 |
| 59 | ENSSSCG00000030921 | APOA1 | 9 | 442.78 | 75.38 | -2.55 | 5.4E-06 | 0.001427 |
| 60 | ENSSSCG00000029796 | KBTBD11 | 15 | 310.44 | 43.22 | -2.84 | 7.1E-06 | 0.001841 |
| 61 | ENSSSCG00000009138 | CFI | 8 | 356.21 | 55.28 | -2.69 | 7.7E-06 | 0.001942 |
| 62 | ENSSSCG00000023498 | - | 6 | 413.92 | 70.35 | -2.56 | 7.7E-06 | 0.001942 |
| 63 | ENSSSCG00000017548 | NGR | 12 | 228.85 | 25.13 | -3.19 | 9.5E-06 | 0.002345 |
| 64 | ENSSSCG00000002368 | LTBP2 | 7 | 1467.64 | 382.92 | -1.94 | 1.06E-05 | 0.002586 |
| 65 | ENSSSCG00000023137 | - | 6 | 339.3 | 53.27 | -2.67 | 1.13E-05 | 0.00272 |
| 66 | ENSSSCG00000024772 | TOMM40 | 6 | 163.18 | 12.06 | -3.76 | 1.21E-05 | 0.002856 |
| 67 | ENSSSCG00000000892 | HAL | 5 | 427.85 | 78.39 | -2.45 | 1.32E-05 | 0.003056 |
| 68 | ENSSSCG00000022758 | PECR | 15 | 900.48 | 217.09 | -2.05 | 1.33E-05 | 0.003056 |
| 69 | ENSSSCG00000006820 | EPS8L3 | 4 | 73.63 | 0 | -6.19 | 1.81E-05 | 0.00409 |
| 70 | ENSSSCG00000006932 | CLCA4 | 4 | 162.19 | 13.07 | -3.63 | 1.95E-05 | 0.004351 |
| 71 | ENSSSCG00000006604 | CRNN | 4 | 215.92 | 25.13 | -3.1 | 2.07E-05 | 0.004556 |
| 72 | ENSSSCG00000026626 | - | 6 | 360.19 | 64.32 | -2.49 | 2.49E-05 | 0.005333 |
| 73 | ENSSSCG00000029613 | SYNM | 1 | 691.53 | 164.83 | -2.07 | 2.72E-05 | 0.005596 |
| 74 | ENSSSCG00000005750 | - | 1 | 311.44 | 51.26 | -2.6 | 2.72E-05 | 0.005596 |
| 75 | ENSSSCG00000029074 | - | 12 | 12679.4 | 4050.31 | -1.65 | 2.92E-05 | 0.005913 |
| 76 | ENSSSCG00000004181 | VNN1 | 1 | 136.32 | 9.04 | -3.91 | 2.98E-05 | 0.005967 |
| 77 | ENSSSCG00000002901 | UPK1A | 6 | 187.06 | 20.1 | -3.22 | 3.34E-05 | 0.006606 |
| 78 | ENSSSCG00000012852 | CDHR5 | 2 | 163.18 | 15.08 | -3.44 | 3.74E-05 | 0.007314 |
| 79 | ENSSSCG00000014031 | - | 2 | 816.9 | 211.06 | -1.95 | 4.05E-05 | 0.007813 |
| 80 | ENSSSCG00000007507 | PCK1 | 17 | 156.22 | 14.07 | -3.47 | 4.47E-05 | 0.008511 |
| 81 | ENSSSCG00000025543 | FABP2 | 8 | 186.07 | 21.11 | -3.14 | 4.72E-05 | 0.008895 |
| 82 | ENSSSCG00000006712 | REG4 | 4 | 66.67 | 0 | -6.05 | 4.78E-05 | 0.008898 |
| 83 | ENSSSCG00000000245 | - | 5 | 242.78 | 36.18 | -2.75 | 5.23E-05 | 0.009618 |
| 84 | ENSSSCG00000015864 | MYO7B | 15 | 98.51 | 4.02 | -4.61 | 6.76E-05 | 0.012147 |
| 85 | ENSSSCG00000015691 | - | 15 | 62.69 | 0 | -5.96 | 8.39E-05 | 0.014757 |
| 86 | ENSSSCG00000009250 | PRKG2 | 8 | 234.82 | 37.19 | -2.66 | 9.52E-05 | 0.016552 |
| 87 | ENSSSCG00000012147 | S100G | X | 72.64 | 1 | -6.18 | 9.64E-05 | 0.016585 |
| 88 | ENSSSCG00000016728 | IGFBP1 | 18 | 162.19 | 18.09 | -3.16 | 0.000103 | 0.017474 |
| 89 | ENSSSCG00000014232 | LOX | 2 | 627.85 | 167.84 | -1.9 | 0.000127 | 0.021165 |
| 90 | ENSSSCG00000003974 | CITED4 | 6 | 69.65 | 1 | -6.11 | 0.000142 | 0.023378 |
| 91 | ENSSSCG00000008461 | ZFP36L2 | 3 | 2627.82 | 885.44 | -1.57 | 0.000145 | 0.023577 |
| 92 | ENSSSCG00000029328 | TNFRSF6 | 2 | 287.56 | 56.28 | -2.35 | 0.000156 | 0.024749 |
| 93 | ENSSSCG00000016746 | NPC1L1 | 18 | 68.66 | 1 | -6.09 | 0.000161 | 0.024981 |
| 94 | ENSSSCG00000002527 | ANKRD9 | 7 | 270.64 | 51.26 | -2.4 | 0.000163 | 0.024981 |
| 95 | ENSSSCG00000030778 | MFI2 | 13 | 68.66 | 1 | -6.09 | 0.000161 | 0.024981 |
| 96 | ENSSSCG00000008557 | EMILIN1 | 3 | 1284.56 | 410.06 | -1.65 | 0.000181 | 0.027476 |
| 97 | ENSSSCG00000026923 | - | 14 | 624.87 | 173.87 | -1.85 | 0.000194 | 0.029261 |
| 98 | ENSSSCG00000011862 | MUC13A | 13 | 55.72 | 0 | -5.79 | 0.000228 | 0.033423 |
| 99 | ENSSSCG00000027593 | - | 12 | 7133.23 | 2602.05 | -1.45 | 0.000236 | 0.033967 |
| 100 | ENSSSCG00000002831 | IRX3 | 6 | 548.25 | 149.75 | -1.87 | 0.000236 | 0.033967 |
| 101 | ENSSSCG00000005930 | SLC45A4 | 4 | 1572.12 | 529.66 | -1.57 | 0.000257 | 0.036561 |
| 102 | ENSSSCG00000002512 | DEGS2 | 7 | 516.41 | 140.71 | -1.88 | 0.000276 | 0.039009 |
| 103 | ENSSSCG00000007530 | PPP1R3D | 17 | 510.44 | 139.7 | -1.87 | 0.000298 | 0.041761 |
| 104 | ENSSSCG00000009558 | F10 | 11 | 71.64 | 2.01 | -5.16 | 0.000303 | 0.042085 |
| 105 | ENSSSCG00000002558 | KIAA0284 | 7 | 5464.6 | 2033.2 | -1.43 | 0.000339 | 0.046683 |
| 106 | ENSSSCG00000016228 | - | 15 | 52.74 | 0 | -5.71 | 0.000352 | 0.048038 |
| 107 | ENSSSCG00000023333 | FCN2 | 1 | 469.64 | 127.64 | -1.88 | 0.000361 | 0.048785 |
| 108 | ENSSSCG00000013614 | CNN1 | 2 | 1988.03 | 714.58 | -1.48 | 0.00043 | 0.05716 |
| 109 | ENSSSCG00000002444 | FBLN5 | 7 | 270.64 | 59.3 | -2.19 | 0.000461 | 0.060819 |
| 110 | ENSSSCG00000026732 | - | 7 | 252.73 | 53.27 | -2.25 | 0.000467 | 0.06101 |
| 111 | ENSSSCG00000010947 | FBP2 | 10 | 160.2 | 24.12 | -2.73 | 0.000514 | 0.066091 |
| 112 | ENSSSCG00000017581 | COL1A1 | 12 | 5194.95 | 1997.02 | -1.38 | 0.000531 | 0.067695 |
| 113 | ENSSSCG00000000385 | SLC39A5 | 5 | 49.75 | 0 | -5.63 | 0.000546 | 0.068511 |
| 114 | ENSSSCG00000009942 | DAO | 14 | 49.75 | 0 | -5.63 | 0.000546 | 0.068511 |
| 115 | ENSSSCG00000005001 | RPL10L | 1 | 299.5 | 71.36 | -2.07 | 0.000571 | 0.07112 |
| 116 | ENSSSCG00000021503 | BCHE | 13 | 158.21 | 24.12 | -2.71 | 0.000582 | 0.071949 |
| 117 | ENSSSCG00000003109 | TMEM160 | 6 | 137.31 | 18.09 | -2.92 | 0.000587 | 0.072018 |
| 118 | ENSSSCG00000014965 | ENDOD1 | 9 | 258.7 | 57.29 | -2.18 | 0.000595 | 0.07238 |
| 119 | ENSSSCG00000017447 | - | 12 | 77.61 | 4.02 | -4.27 | 0.000635 | 0.075565 |
| 120 | ENSSSCG00000023212 | CPI17 | 6 | 189.05 | 34.17 | -2.47 | 0.00063 | 0.075565 |
| 121 | ENSSSCG00000005485 | AMBP | 1 | 48.76 | 0 | -5.6 | 0.000632 | 0.075565 |
| 122 | ENSSSCG00000004632 | GLDN | 1 | 279.6 | 66.33 | -2.08 | 0.000714 | 0.083676 |
| 123 | ENSSSCG00000007816 | IL21R | 3 | 348.25 | 92.46 | -1.91 | 0.000768 | 0.088725 |
| 124 | ENSSSCG00000000664 | A2ML1 | 5 | 159.2 | 26.13 | -2.61 | 0.00082 | 0.092984 |
| 125 | ENSSSCG00000023215 | MAOB | X | 253.73 | 58.29 | -2.12 | 0.000823 | 0.092984 |
| 126 | ENSSSCG00000012536 | PLP1 | X | 46.77 | 0 | -5.54 | 0.000848 | 0.095178 |
| 127 | ENSSSCG00000006029 | NUDCD1 | 4 | 45.77 | 0 | -5.51 | 0.000983 | 0.108802 |
| 128 | ENSSSCG00000012236 | OTC | X | 45.77 | 0 | -5.51 | 0.000983 | 0.108802 |
| 129 | ENSSSCG00000007574 | SDK1 | 3 | 310.44 | 83.42 | -1.9 | 0.001219 | 0.130291 |
| 130 | ENSSSCG00000012699 | FHL1C | X | 1582.07 | 612.07 | -1.37 | 0.001274 | 0.135309 |
| 131 | ENSSSCG00000009132 | ENPEP | 8 | 228.85 | 53.27 | -2.1 | 0.001314 | 0.138628 |
| 132 | ENSSSCG00000028878 | BCAR1 | 6 | 1697.49 | 668.35 | -1.34 | 0.001447 | 0.150653 |
| 133 | ENSSSCG00000022258 | - | 17 | 176.12 | 35.18 | -2.32 | 0.001478 | 0.152793 |
| 134 | ENSSSCG00000001785 | MESDC1 | 7 | 540.29 | 179.9 | -1.59 | 0.001538 | 0.158019 |
| 135 | ENSSSCG00000022553 | TNRC18 | 3 | 310.44 | 86.43 | -1.84 | 0.001577 | 0.160936 |
| 136 | ENSSSCG00000007927 | PPL | 3 | 5934.24 | 2502.55 | -1.25 | 0.00161 | 0.163309 |
| 137 | ENSSSCG00000001873 | CSPG4 | 7 | 1138.29 | 436.19 | -1.38 | 0.001667 | 0.167443 |
| 138 | ENSSSCG00000021610 | CHPF | 15 | 1809.92 | 725.64 | -1.32 | 0.001672 | 0.167443 |
| 139 | ENSSSCG00000002265 | FAM174B | 7 | 179.1 | 37.19 | -2.27 | 0.001703 | 0.168598 |
| 140 | ENSSSCG00000013579 | CD209 | 2 | 884.56 | 328.65 | -1.43 | 0.001727 | 0.169657 |
| 141 | ENSSSCG00000002524 | AMN | 7 | 41.79 | 0 | -5.38 | 0.001784 | 0.174204 |
| 142 | ENSSSCG00000017012 | SLIT3 | 16 | 1362.17 | 536.69 | -1.34 | 0.001815 | 0.176197 |
| 143 | ENSSSCG00000011850 | MUC4 | 13 | 3788 | 1596 | -1.25 | 0.001867 | 0.180129 |
| 144 | ENSSSCG00000009722 | SPOCK3 | 14 | 92.54 | 10.05 | -3.2 | 0.001955 | 0.186011 |
| 145 | ENSSSCG00000025924 | IGFBP5 | 15 | 839.79 | 313.57 | -1.42 | 0.001963 | 0.186011 |
| 146 | ENSSSCG00000022280 | DACT3 | 6 | 193.03 | 43.22 | -2.16 | 0.001946 | 0.186011 |
| 147 | ENSSSCG00000024018 | SLC16A3 | 12 | 213.93 | 51.26 | -2.06 | 0.001996 | 0.187918 |
| 148 | ENSSSCG00000025423 | KCNK5 | 7 | 1897.48 | 780.92 | -1.28 | 0.002148 | 0.198261 |
| 149 | ENSSSCG00000015404 | - | 9 | 357.21 | 109.55 | -1.71 | 0.002155 | 0.198261 |
| 150 | ENSSSCG00000009672 | SCARA5 | 14 | 455.71 | 150.76 | -1.6 | 0.002138 | 0.198261 |
| 151 | ENSSSCG00000005845 | TOR4A | 1 | 79.6 | 7.04 | -3.5 | 0.002173 | 0.198681 |
| 152 | ENSSSCG00000017192 | EVPL | 12 | 7741.18 | 3375.93 | -1.2 | 0.002254 | 0.204928 |
| 153 | ENSSSCG00000013088 | LRRC10B | 2 | 107.46 | 15.08 | -2.83 | 0.002408 | 0.217734 |
| 154 | ENSSSCG00000026610 | RNF10 | 14 | 625.86 | 229.15 | -1.45 | 0.002669 | 0.239911 |
| 155 | ENSSSCG00000007485 | BCAS1 | 17 | 761.18 | 289.45 | -1.39 | 0.002721 | 0.243176 |
| 156 | ENSSSCG00000009513 | SLC15A1 | 11 | 4680.53 | 2062.34 | -1.18 | 0.002898 | 0.248255 |
| 157 | ENSSSCG00000002392 | IRF2BPL | 7 | 1389.03 | 571.87 | -1.28 | 0.002826 | 0.248255 |
| 158 | ENSSSCG00000025805 | - | 3 | 80.6 | 8.04 | -3.33 | 0.002866 | 0.248255 |
| 159 | ENSSSCG00000029675 | MMP8 | 9 | 91.54 | 11.05 | -3.05 | 0.002885 | 0.248255 |
| 160 | ENSSSCG00000010792 | PRAP1 | 14 | 46.77 | 1 | -5.54 | 0.002913 | 0.248255 |
| 161 | ENSSSCG00000026978 | ROS1 | 1 | 46.77 | 1 | -5.54 | 0.002913 | 0.248255 |
| 162 | ENSSSCG00000025578 | ALDH1A2 | 1 | 135.32 | 25.13 | -2.43 | 0.002919 | 0.248255 |
| 163 | ENSSSCG00000005094 | TMEM30B | 1 | 2730.31 | 1177.91 | -1.21 | 0.002859 | 0.248255 |
| 164 | ENSSSCG00000006312 | GPA33 | 4 | 116.42 | 19.1 | -2.61 | 0.003139 | 0.265603 |
| 165 | ENSSSCG00000000211 | AQP5 | 5 | 2358.17 | 1021.12 | -1.21 | 0.003231 | 0.271918 |
| 166 | ENSSSCG00000025510 | SLC13A2 | 18 | 37.81 | 0 | -5.23 | 0.003256 | 0.272125 |
| 167 | ENSSSCG00000017885 | SMTNL2 | 12 | 244.77 | 69.35 | -1.82 | 0.003602 | 0.295572 |
| 168 | ENSSSCG00000010322 | ZNF503 | 14 | 299.5 | 92.46 | -1.7 | 0.003606 | 0.295572 |
| 169 | ENSSSCG00000028460 | S1PR5 | 2 | 229.85 | 63.32 | -1.86 | 0.003651 | 0.297145 |
| 170 | ENSSSCG00000013501 | CREB3L3 | 2 | 81.59 | 9.04 | -3.17 | 0.003662 | 0.297145 |
| 171 | ENSSSCG00000006693 | PDZK1 | 4 | 36.81 | 0 | -5.19 | 0.003789 | 0.305841 |
| 172 | ENSSSCG00000007993 | SOLH | 3 | 1477.59 | 630.16 | -1.23 | 0.003842 | 0.308572 |
| 173 | ENSSSCG00000004735 | - | 1 | 335.32 | 109.55 | -1.61 | 0.004021 | 0.321278 |
| 174 | ENSSSCG00000022317 | SLC38A10 | 12 | 1646.74 | 712.57 | -1.21 | 0.004086 | 0.32488 |
| 175 | ENSSSCG00000005967 | FAM84B | 4 | 701.48 | 275.38 | -1.35 | 0.004123 | 0.326125 |
| 176 | ENSSSCG00000031023 | CD24 | 1 | 9899.35 | 4579.97 | -1.11 | 0.004291 | 0.336089 |
| 177 | ENSSSCG00000025993 | SGPP2 | 15 | 1316.4 | 562.82 | -1.23 | 0.004362 | 0.339983 |
| 178 | ENSSSCG00000005380 | - | 1 | 741.28 | 296.49 | -1.32 | 0.004495 | 0.346897 |
| 179 | ENSSSCG00000000050 | - | 5 | 848.74 | 346.74 | -1.29 | 0.004543 | 0.347746 |
| 180 | ENSSSCG00000011355 | COL7A1 | 13 | 655.71 | 257.29 | -1.35 | 0.00455 | 0.347746 |
| 181 | ENSSSCG00000012001 | ROBO1 | 13 | 230.84 | 66.33 | -1.8 | 0.004645 | 0.353319 |
| 182 | ENSSSCG00000007369 | JPH2 | 17 | 213.93 | 59.3 | -1.85 | 0.004681 | 0.354306 |
| 183 | ENSSSCG00000005751 | COL5A1 | 1 | 4951.17 | 2282.45 | -1.12 | 0.004736 | 0.356831 |
| 184 | ENSSSCG00000003603 | COL16A1 | 6 | 449.75 | 163.82 | -1.46 | 0.004762 | 0.357056 |
| 185 | ENSSSCG00000012394 | GJB1 | X | 942.27 | 392.97 | -1.26 | 0.004845 | 0.359846 |
| 186 | ENSSSCG00000022645 | H2AFX | 9 | 250.74 | 75.38 | -1.73 | 0.004867 | 0.359846 |
| 187 | ENSSSCG00000028623 | RARRES1 | 13 | 250.74 | 75.38 | -1.73 | 0.004867 | 0.359846 |
| 188 | ENSSSCG00000004012 | THBS2 | 1 | 1443.76 | 630.16 | -1.2 | 0.00495 | 0.364156 |
| 189 | ENSSSCG00000021173 | - | 1 | 572.13 | 221.11 | -1.37 | 0.004972 | 0.364156 |
| 190 | ENSSSCG00000012832 | MXRA5 | X | 19938 | 9414.21 | -1.08 | 0.005035 | 0.365167 |
| 191 | ENSSSCG00000025146 | - | 6 | 1927.33 | 860.32 | -1.16 | 0.005044 | 0.365167 |
| 192 | ENSSSCG00000012576 | CHRDL1 | X | 53.73 | 3.02 | -4.16 | 0.005094 | 0.366295 |
| 193 | ENSSSCG00000023675 | - | 10 | 73.63 | 8.04 | -3.19 | 0.005394 | 0.386132 |
| 194 | ENSSSCG00000023784 | - | 9 | 965.16 | 410.06 | -1.23 | 0.005608 | 0.396096 |
| 195 | ENSSSCG00000012982 | SIPA1 | 2 | 323.38 | 109.55 | -1.56 | 0.005641 | 0.396672 |
| 196 | ENSSSCG00000016609 | SLC13A1 | 18 | 41.79 | 1 | -5.38 | 0.00569 | 0.398322 |
| 197 | ENSSSCG00000010527 | MARVELD1 | 14 | 197.01 | 54.27 | -1.86 | 0.005743 | 0.400274 |
| 198 | ENSSSCG00000005610 | SLC2A8 | 1 | 709.44 | 291.46 | -1.28 | 0.006055 | 0.418313 |
| 199 | ENSSSCG00000021753 | - | 18 | 64.68 | 6.03 | -3.42 | 0.006223 | 0.428062 |
| 200 | ENSSSCG00000021015 | - | 12 | 5607.88 | 2662.35 | -1.07 | 0.006331 | 0.430809 |
| 201 | ENSSSCG00000008550 | SLC5A6 | 3 | 436.81 | 163.82 | -1.41 | 0.006305 | 0.430809 |
| 202 | ENSSSCG00000010109 | SCARF2 | 14 | 312.43 | 106.53 | -1.55 | 0.006377 | 0.430809 |
| 203 | ENSSSCG00000027275 | HHLA2 | 13 | 51.74 | 3.02 | -4.1 | 0.006399 | 0.430809 |
| 204 | ENSSSCG00000013079 | DAGLA | 2 | 523.38 | 205.03 | -1.35 | 0.006441 | 0.431767 |
| 205 | ENSSSCG00000014255 | SLC12A2 | 2 | 876.6 | 374.88 | -1.23 | 0.006633 | 0.442791 |
| 206 | ENSSSCG00000009545 | COL4A2 | 11 | 28634.4 | 13917.8 | -1.04 | 0.006822 | 0.448552 |
| 207 | ENSSSCG00000024812 | - | 3 | 1063.67 | 466.34 | -1.19 | 0.006795 | 0.448552 |
| 208 | ENSSSCG00000016034 | COL3A1 | 15 | 64826.9 | 31585.4 | -1.04 | 0.006833 | 0.448552 |
| 209 | ENSSSCG00000027302 | - | 2 | 1073.62 | 471.36 | -1.19 | 0.006823 | 0.448552 |
| 210 | ENSSSCG00000027956 | SSH3 | 2 | 761.18 | 320.61 | -1.25 | 0.006862 | 0.448606 |
| 211 | ENSSSCG00000010479 | RBP4 | 14 | 729.34 | 305.53 | -1.26 | 0.006911 | 0.449981 |
| 212 | ENSSSCG00000005843 | LCN15 | 1 | 32.84 | 0 | -5.03 | 0.006973 | 0.450294 |
| 213 | ENSSSCG00000022289 | PCDH1 | 2 | 3083.54 | 1453.29 | -1.09 | 0.007004 | 0.45045 |
| 214 | ENSSSCG00000014625 | TRIM3 | 9 | 433.82 | 164.83 | -1.4 | 0.007044 | 0.451165 |
| 215 | ENSSSCG00000005177 | SH3GL2 | 1 | 273.63 | 90.45 | -1.6 | 0.007079 | 0.451589 |
| 216 | ENSSSCG00000008004 | FAM173A | 3 | 299.5 | 102.51 | -1.55 | 0.007194 | 0.455291 |
| 217 | ENSSSCG00000008664 | FAM84A | 3 | 2112.41 | 982.93 | -1.1 | 0.007301 | 0.460165 |
| 218 | ENSSSCG00000029421 | - | 1 | 507.45 | 202.01 | -1.33 | 0.007695 | 0.479312 |
| 219 | ENSSSCG00000007583 | - | 3 | 82.59 | 12.06 | -2.78 | 0.007792 | 0.482051 |
| 220 | ENSSSCG00000010120 | C22ORF39 | 14 | 154.23 | 39.2 | -1.98 | 0.007825 | 0.482051 |
| 221 | ENSSSCG00000013896 | MPV17L2 | 2 | 320.39 | 113.57 | -1.5 | 0.007831 | 0.482051 |
| 222 | ENSSSCG00000016600 | TMEM229A | 18 | 1118.39 | 502.52 | -1.15 | 0.008144 | 0.497497 |
| 223 | ENSSSCG00000013377 | USH1C | 2 | 31.84 | 0 | -4.99 | 0.00813 | 0.497497 |
| 224 | ENSSSCG00000023404 | FAM89A | 14 | 487.56 | 194.98 | -1.32 | 0.008506 | 0.515583 |
| 225 | ENSSSCG00000026422 | GPER | 3 | 130.35 | 30.15 | -2.11 | 0.008562 | 0.517026 |
| 226 | ENSSSCG00000002545 | ZFYVE21 | 7 | 101.49 | 19.1 | -2.41 | 0.008626 | 0.518894 |
| 227 | ENSSSCG00000011963 | TOMM70A | 13 | 569.15 | 235.18 | -1.28 | 0.008671 | 0.519635 |
| 228 | ENSSSCG00000012083 | RIPK4 | 13 | 3121.35 | 1503.54 | -1.05 | 0.008728 | 0.52105 |
| 229 | ENSSSCG00000003514 | HSPG2 | 6 | 7683.47 | 3790.01 | -1.02 | 0.008985 | 0.534405 |
| 230 | ENSSSCG00000025632 | - | 4 | 141.29 | 35.18 | -2.01 | 0.009221 | 0.54042 |
| 231 | ENSSSCG00000008695 | MFSD10 | 8 | 588.05 | 246.24 | -1.26 | 0.009205 | 0.54042 |
| 232 | ENSSSCG00000015326 | COL1A2 | 9 | 57464.8 | 28771.3 | -0.998 | 0.009223 | 0.54042 |
| 233 | ENSSSCG00000003371 | GPR153 | 6 | 1270.63 | 584.93 | -1.12 | 0.009169 | 0.54042 |
| 234 | ENSSSCG00000021991 | - | 13 | 445.76 | 176.89 | -1.33 | 0.009296 | 0.542717 |
| 235 | ENSSSCG00000008701 | LREAP1 | 8 | 5730.26 | 2823.16 | -1.02 | 0.009338 | 0.543128 |
| 236 | ENSSSCG00000023423 | FAM100B | 12 | 807.95 | 355.78 | -1.18 | 0.009412 | 0.545432 |
| 237 | ENSSSCG00000013338 | SLC5A12 | 2 | 30.84 | 0 | -4.94 | 0.009483 | 0.54755 |
| 238 | ENSSSCG00000016784 | ANKH | 16 | 2273.6 | 1090.47 | -1.06 | 0.00952 | 0.54769 |
| 239 | ENSSSCG00000008722 | - | 8 | 340.29 | 126.64 | -1.43 | 0.009612 | 0.550986 |
| 240 | ENSSSCG00000002696 | VAT1L | 6 | 102.49 | 20.1 | -2.35 | 0.009668 | 0.552217 |
| 241 | ENSSSCG00000017459 | KRT20 | 12 | 37.81 | 1 | -5.23 | 0.00974 | 0.554311 |
